# Current State of Community-Driven Radiological AI Deployment in Medical Imaging

**DOI:** 10.2196/55833

**Published:** 2024-12-09

**Authors:** Vikash Gupta, Barbaros Erdal, Carolina Ramirez, Ralf Floca, Bradley Genereaux, Sidney Bryson, Christopher Bridge, Jens Kleesiek, Felix Nensa, Rickmer Braren, Khaled Younis, Tobias Penzkofer, Andreas Michael Bucher, Ming Melvin Qin, Gigon Bae, Hyeonhoon Lee, M Jorge Cardoso, Sebastien Ourselin, Eric Kerfoot, Rahul Choudhury, Richard D White, Tessa Cook, David Bericat, Matthew Lungren, Risto Haukioja, Haris Shuaib

**Affiliations:** 1 Mayo Clinic Jacksonville, FL United States; 2 University of California San Francisco, CA United States; 3 German Cancer Research Center (DKFZ) Heidelberg Germany; 4 Nvidia Inc Santa Clara, CA United States; 5 Massachusetts General Hospital Boston, MA United States; 6 Institute for AI in Medicine University Medicine Essen Essen Germany; 7 Institute for Radiology School of Medicine Technical University Munich Germany; 8 MedAiConsult Cleveland, OH United States; 9 Department of Radiology Charité – Universitätsmedizin Berlin Germany; 10 Berlin Institute of Health Berlin Germany; 11 University Hospital Frankfurt/Main Frankfurt Germany; 12 Biomedical Research Institute Seoul National University Hospital Seoul Republic of Korea; 13 School of Biomedical Engineering & Imaging Sciences King's College London United Kingdom; 14 Perelman School of Medicine University of Pennsylvania Philadelphia, PA United States; 15 Microsoft Inc Palo Alto, CA United States; 16 Clinical Scientific Computing, Medical Physics Newton's Tree London United Kingdom

**Keywords:** radiology, open-source, radiology in practice, deep learning, artificial intelligence, imaging informatics, clinical deployment, imaging, medical informatics, workflow, operation, implementation, adoption, taxonomy, use case, model, integration, machine learning, mobile phone

## Abstract

Artificial intelligence (AI) has become commonplace in solving routine everyday tasks. Because of the exponential growth in medical imaging data volume and complexity, the workload on radiologists is steadily increasing. AI has been shown to improve efficiency in medical image generation, processing, and interpretation, and various such AI models have been developed across research laboratories worldwide. However, very few of these, if any, find their way into routine clinical use, a discrepancy that reflects the divide between AI research and successful AI translation. The goal of this paper is to give an overview of the intersection of AI and medical imaging landscapes. We also want to inform the readers about the importance of using standards in their radiology workflow and the challenges associated with deploying AI models in the clinical workflow. The main focus of this paper is to examine the existing condition of radiology workflow and identify the challenges hindering the implementation of AI in hospital settings. This report reflects extensive weekly discussions and practical problem-solving expertise accumulated over multiple years by industry experts, imaging informatics professionals, research scientists, and clinicians. To gain a deeper understanding of the requirements for deploying AI models, we introduce a taxonomy of AI use cases, supplemented by real-world instances of AI model integration within hospitals. We will also explain how the need for AI integration in radiology can be addressed using the Medical Open Network for AI (MONAI). MONAI is an open-source consortium for providing reproducible deep learning solutions and integration tools for radiology practice in hospitals.

## Introduction

There are multiple well-recognized applications of artificial intelligence (AI) in health care. Radiology has become the leading focus of health care–AI research, and there has been an exponential rise in the number of related publications and AI-enabled devices approved by the US Food and Drug Administration (FDA) [1]. However, the use of AI in a clinical workflow has received little attention from the industry and research communities. Ebrahimian et al [2] found that 59 out of 118 FDA-approved AI/machine learning models are solely image-processing focused.

Multiple commercial entities offer a range of AI solutions specifically targeted toward hospitals. These entities typically collaborate with the hospital’s imaging informatics team to facilitate the implementation of their solutions within the organization. This collaboration involves working closely with the hospital’s experts to integrate and deploy the AI solutions effectively. By partnering with the hospital’s imaging informatics team, these commercial entities ensure seamless integration of their AI solutions into the existing infrastructure and workflows of the hospital, thereby optimizing the impact and usability of their offerings within the health care setting.

In an ideal scenario, when a radiologist identifies a specific need for an AI model that can enhance their daily workflow, they should have the ability to label their own data and train a customized model accordingly. This approach is often reflected in research papers published within academic and research communities [3-8]. However, the intention of this paper is to accelerate the progress of AI research and bring it to the forefront of practical implementation as quickly as possible. By focusing on key aspects of AI research and deployment in the health care domain [9], this paper aims to facilitate the adoption of AI technologies in real-world scenarios, empowering radiologists and health care professionals to leverage AI’s potential for improved efficiency and patient care.

By highlighting the barriers [10,11] associated with deploying AI models and providing practical ways to overcome them, we aim to offer a comprehensive understanding of the imaging informatics landscape. Through our examples and analysis, we hope to equip AI researchers and hospitals with the necessary knowledge and tools to build and deploy their own AI models using their own data. Our emphasis is on using open-source, community-driven tools for AI model deployment, which enables researchers to leverage the collective expertise of the wider scientific community and ultimately drive innovation in health care.

The radiology and imaging informatics landscape [12] is so diverse because of differences in implementations across different institutions. One of the ways to ease the implementation is by building our tools compatible with existing imaging informatics standards like Digital Imaging and Communication in Medicine (DICOM) [13], Health Level 7 (HL7), and Fast Health Care Interoperability Resources [14]. By aligning our tools with these established standards, we can ensure seamless integration with existing health care systems and infrastructure. This compatibility enables interoperability and data exchange between different systems, making it easier for hospitals and health care facilities to adopt and use AI solutions without disrupting their current workflows. We explain the changes in the radiology workflow introduced by the usage of AI and deep learning. In that context, we also provide the solutions available through the Medical Open Network for AI (MONAI) working group. MONAI is an open-source consortium comprised of health care and informatics professionals as well as clinicians and researchers. This paper and the methodologies discussed in this paper are a result of weekly discussions in the different MONAI working groups.

We also present a taxonomy of AI-based solution scenarios. Categorizing the AI use case in such a manner helps to have a broad categorization of AI tools and deployment scenarios. This taxonomy serves as a valuable resource for health care professionals, AI researchers, and hospitals, as it facilitates a systematic understanding of different AI use cases and their potential benefits. It enables stakeholders to explore and evaluate various AI solutions within specific domains or applications, ultimately aiding in the selection and implementation of the most suitable AI tools for their unique requirements. Our goal in this paper is that the reader will be able to see how AI tools can be deployed using open-source tools developed in MONAI Deploy.

## Barriers to Incorporating Imaging AI into Clinical Practice

Generic AI applications (eg, news feeds and shopping recommendations) have become ubiquitous in our lives; using a centralized infrastructure, the associated user experience adapts to include such AI applications [15]. Related novel support tools and vocabulary (eg, tap and drag) have entered our common vernacular only in the last decade [16]. This behavior modification was driven largely by smartphone and computer manufacturers.

On the other hand, such centralized operations are ineffective in medical facilities and health care systems, primarily due to the nonuniform nature of deployment infrastructures. This inhomogeneity is related to differences in acquisition protocols, radiologist workflows, data management procedures, and IT architectures [17]. The evolution of imaging AI workflows is complicated, as different components of this system progress at different paces and in different directions yet are required to be always interconnected.

AI processing must seamlessly integrate with existing clinical workflows, where imaging impacts downstream decisions, such as surgery, interventions, and therapies [18]. New AI-based workflows should either not interfere with established routine practices or significantly improve their efficiency such that learning a new workflow becomes desirable rather than burdensome. Radiologists must be trained in the use of AI-augmented processes to further maximize the likelihood that they will use the AI and realize its benefits. AI tools should have configurable end points that can be integrated into diverse health care system infrastructures across multiple vendors and protocols. An optimally deployed AI should ideally be indistinguishable from the existing IT infrastructure. Nevertheless, the introduction of such tools requires the fostering of trust among the users (eg, radiologists, technologists, and other members of the care team) and beneficiaries (eg, patients and referring clinicians). In addition, AI workflows should support data and workflow standards to increase the likelihood of systems interoperability and seamless integration [19,20].

## Intersystem Communication and Interoperability

### Overview

Medical facilities and health care systems rely on many software applications to meet diverse clinical, research, educational, and business needs. Such applications track patient care across disciplines; support inpatient monitoring; and facilitate procedure scheduling, billing, and much more. These applications must be interoperable so that information is not repeatedly entered, managed, or siloed in a single system or department. DICOM and HL7 have existed for more than 3 decades to facilitate the exchange of imaging and health data, respectively.

Integrating into the Healthcare Enterprise (IHE) has defined profiles that organize and leverage the aforementioned integration capabilities, containing specific information about diverse clinical needs. IHE profiles should guide the development of AI applications and the definition of integration points and workflows. More recently, Fast Health Care Interoperability Resources–based profiles have enabled semantically interoperable exchange of machine-readable data [21]. IHE profiles were consulted while creating the MONAI Deploy tools.

### MONAI Deploy

MONAI is an open-source community of researchers, clinicians, and imaging informatics professionals. Within MONAI, there are several working groups; one of them being MONAI Deploy. It aims to become the de facto standard for developing packaging, testing, and deploying and running medical AI applications in clinical production. The goal of MONAI Deploy is to accelerate the development of medical imaging AI inference applications with DICOM imaging network integration.

### AI Integration Points in Health Care Infrastructure

Understanding the systems that make up a given workflow is the first step when considering AI integration touchpoints. The interoperability of health care systems is critical in the delivery of good patient care, but adding new applications into a complex workflow can be challenging if the interfaces between systems are not well understood.

**Figure 1:**
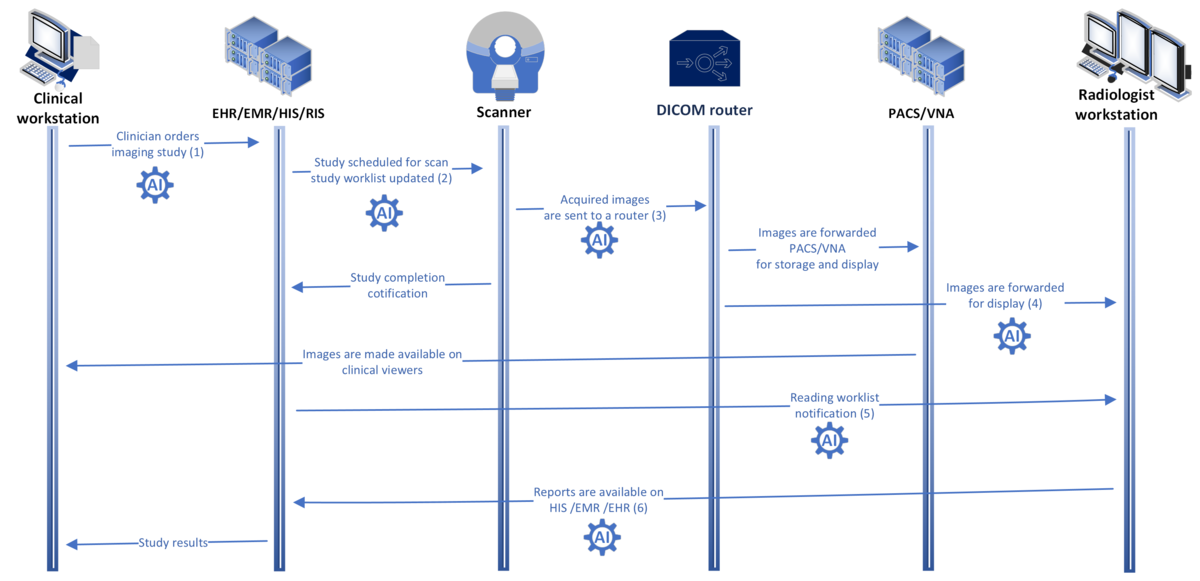
The figure shows a basic AI-enabled radiology workflow, modeled after integrating the health care enterprise scheduled workflow that shows an order being generated, a patient being imaged, images being evaluated by a radiologist, and a report being generated and sent back to the ordering clinician for review. AI: artificial intelligence; DICOM: digital imaging and communications in medicine; EHR: electronic health record; EMR: electronic medical record; HIS: hospital information system; PACS: picture archiving and communication systems; RIS: radiology information system; VNA: vendor neutral archive.

AI integration into the workflow must leverage interoperability with existing systems to be effective. The IHE AI in imaging white paper [22], which references AI workflow for imaging [23] and AI results [24], describes the steps and boundaries that should be considered. Some examples of using AI in clinical workflows can be seen in Figure 1 [16], and their relation to AI workflow for imaging and AI results can be found in Table 1. The table also shows how these capabilities were achieved using MONAI Deploy tools.

While there are early stages of cloud-based Picture Archiving and Communication Systems solutions, for most health care institutions today, the emphasis remains on-premises deployments. An on-premise deployment refers to the computing model when the organization’s IT infrastructure is located in its own physical space. In the AI space, there are many vendor examples that use cloud-based implementations; however, since most Picture Archiving and Communication Systems are maintained by the corresponding hospitals, this brings hybrid implementations into the picture. In these scenarios, vendors deliver solutions through either client systems (which connect to their cloud servers) or by delivering their results to destination workstations through proxy servers operating across firewalls. MONAI Deploy [25] enables the production of containers (“Docker containers”), which can be executed either on premises or on the cloud as needed based on the given site’s architecture. For efficient task and resource management, MONAI Deploy offers a task management system that can be used, if required, to manage containers. For facilitating standards-based (eg, DICOM and HL7) communication, MONAI Deploy also offers an informatics gateway solution.

**Table 1:** Common workflow steps as highlighted by participating institutions versus AI workflow– and AI results–described boundaries.

Steps	Common workflow theme	AIW-I^a^ and AIR^b^ described boundaries	MONAI^c^ capabilities
1	When a clinician orders an imaging examination in the HIS^d^ or RIS^e^, they may be guided by a CDSS^f^ to ensure its appropriateness. Depending on the clinical setting, the order may contain a clinical-status priority code (eg, “stat”).	Make recommendations as to the types of procedures that should be ordered, based on the patient’s condition and record	—^g^
2	Once the patient examination is scheduled for a date and location, an entry is created on the “study worklist” of the scanner (or another imaging device). In some instances, an entry is also created on a “protocoling worklist,” where a radiologist determines the specific imaging techniques to be used (eg, scanning details, contrast-agent type, amount, or administration route) during the diagnostic imaging study or image-directed procedure.	Make recommendations on the type of protocol to be used on the scanner	—
3	Once the examination is completed, images are reconstructed into a human-interpretable format and sent to a DICOM^h^-router to be forwarded to the appropriate destinations, including a PACS^i^ or VNA^j^ for management or storage. Once the organized images (original or postprocessed) are ready to be evaluated by the radiologist, the examination description appears on the radiologist’s “reading worklist.”	Postprocess the image, identify QA^k^ issues before the patient leaves the department, and prepare classifications and segmentations in advance of the radiologist’s evaluation	Has workflow management functionalities to handle imaging studies, AI^l^ algorithm execution, and AIR forwarding functionalities compatible with standards such as DICOM, HL7^m^, etc.
4	Radiologists assess the examination images on their diagnostic viewer and dictate their interpretation (typically into a voice recognition system).	Include insights alongside the images in the radiologist’s display	Can produce AI inference results compatible with current transmission and display standards.
5	The dictated report is sent to the HIS/RIS. If actionable critical or noncritical findings are identified, radiologists may invoke additional workflows to alert the ordering clinician, along with issuing the final examination report.	Include emergent insights for consideration by the ordering physician	It can be used to repackage the inference in a DICOM-SEG^n^, SR^o^, or HL7, etc, to be sent to desired destinations through reports or alerts.
6	Final examination reports become available in the HIS or EHR^p^, along with the images in the PACS or clinical viewers.	Prepopulate the radiologist’s report with draft insights to be considered by the radiologist	It can be used to produce and send structured reports to desired destinations.

^a^AIW-I: artificial intelligence workflow for imaging.

^b^AIR: artificial intelligence results.

^c^MONAI: Medical Open Network for Artificial Intelligence.

^d^HIS: Hospital Information System.

^e^RIS: Radiology Information System.

^f^CDSS: Clinical Decision Support System.

^g^Not available.

^h^DICOM: Digital Imaging and Communication in Medicine.

^i^PACS: Picture Archiving and Communication Systems.

^j^VNA: Vendor Neutral Archive.

^k^QA: quality assurance.

^l^AI: artificial intelligence.

^m^HL7: Health Level 7.

^n^DICOM-SEG: Digital Imaging and Communication in Medicine Segments.

^o^SR: structured report.

^p^EHR: electronic health record.

## A Taxonomy of Imaging AI Use Cases

### Overview

AI deployment in radiology encompasses a wide spectrum, considering both the implementation process and the user experience. The outputs generated by AI models can vary significantly, including plain text outputs such as probability values for classification, segmentation masks, signals to prompt action (eg, patient prioritization), or coordinates indicating the location of detected abnormalities or malignancies. In addition, radiological images themselves can be either 2D or 3D, following different DICOM protocols.

Given the diverse nature of AI use cases in radiology, it is logical and helpful to categorize them for ease of discussion and analysis. These categories, however, should not be considered rigid or fixed, as the field of AI in radiology continues to evolve rapidly. This categorization approach allows for a more systematic exploration of AI deployment scenarios in radiology, facilitating knowledge sharing, collaboration, and the identification of common patterns or trends across different use cases. It also enables researchers and clinicians to discuss specific categories in more depth, share insights, and learn from experiences in similar contexts.

### Emergency Diagnostics

Time is of the essence in some emergency situations (eg, stroke, cardiac arrest, and trauma), when the emergency department must await imaging results to determine the most suitable next steps in patient care. For example, when a patient shows signs of head trauma, the emergency department often orders emergency computed tomography (CT) imaging to detect possible skull fractures and brain hemorrhage [26]; this could be expedited with imaging AI. The alternative would be to wait for radiologists to go through a reading worklist in a nonprioritized sequential order before realizing which are the most emergent cases.

### Integrated Diagnostic Radiology Examination Planning

Currently, the scheduling of a new imaging examination and the review of previous imaging data for the protocol of that examination are typically performed separately and asynchronously by different participants (schedulers, technologists vs physicians, respectively). With the help of imaging AI, both scheduling and review of diagnostic radiology examination planning could be made to be both concurrent and complementary, leading to optimized prospective examination planning, including enhanced scanner and protocol selection. Hence, any guidance on safety measures (eg, handling of magnetic resonance imaging–unsafe devices and proper use of contrast agents), guidance on needed imaging sequences or appropriate scanning hardware (eg, a 3 Tesla vs a 1.5 Tesla Magnet), as well as scheduling for appropriate time slots can be recommended.

### Opportunistic Screening

The term “opportunistic screening” refers to the application of imaging AI technology to pixel data to improve wellness, prevent disease, assess risk, or detect asymptomatic disease. The opportunistic concept focuses on enhanced screening for silent conditions or risk factors that are incidental to the primary indication for the imaging examination. Among the most promising opportunistic screening use cases is the training of a model to detect and quantify coronary artery calcium for cardiovascular risk stratification on routine chest CTs performed with high frequency for noncardiovascular reasons (eg, interstitial lung disease, trauma, and low-dose lung nodule screening) [27]. Quantification of coronary artery calcium on non–cardiac-gated chest CTs is complicated by motion-related artifacts and protocol heterogeneity.

Another relevant use case for opportunistic screening is CT-based body composition analysis, which quantifies liver fat, organ volumes, and muscle loss, among other characteristics [28-30].

### Interactive Diagnostics During Image Interpretation

Radiologists often consult their radiology colleagues for diagnostic mediation when interpreting challenging or ambiguous cases (eg, possible cancers [31,32], subtle fractures [33-35], coronary stenosis [4,36], and cancer detection in dense breasts [37,38]). In the process, the work of both radiologists is interrupted, and department workflow is slowed. The alternative of not seeking such peer consultation, but rather ordering additional diagnostic evaluations, can prolong patient assessment, delay treatment initiation, and lead to additional risks and expenses. Hence, an AI-based “second-opinion” support system could be highly beneficial to both radiologists and patients.

### Large-Scale AI Model Validation

Radiologists increasingly collaborate with AI researchers to identify new areas of research and model development. Such collaborations yield large-scale statistical analyses to identify trends and gather insights about disease progression and risk factors; this can stimulate novel radiomic or radiogenomic-based diagnostics. For example, there are ongoing efforts in the neuroimaging community to identify imaging biomarkers for Alzheimer disease [39,40] and Parkinson disease [41]. Consortiums such as the Alzheimer’s Disease Neuroimaging Initiative, Parkinson’s Progression Markers Initiative, UK Biobank, and The Cancer Imaging Archive are facilitating the development of AI for imaging-driven precision medicine.

Large-scale AI-model validation by rigorous evaluation and standardized reporting on AI applications in health care is essential. As stated by Panch et al [42], there has long been a gap between the expectation created from impressive small-scale research evaluations on medical-AI applications and a relative sparsity of distribution of similar applications in the real-world clinical pathways. There are numerous reasons to explain this gap, but a very important point is the lack of well-established common infrastructures to assist in the training, evaluation, and distribution of AI models between health care institutions. These kinds of infrastructures can only be derived from large-scale collaborations between the different components as discussed in the following sections.

## Real-World Use Cases

### Overview

The authors of this paper have presented several use cases that originated within their respective institutions. These institutions include renowned organizations such as the Mayo Clinic, University of California San Francisco, University of Pennsylvania, Massachusetts General Brigham Hospital, Guy’s and St Thomas Hospital, National Health System, and German Cancer Research Center. The AI tools developed by these institutions were initially part of original research projects and subsequently integrated into clinical practice. For detailed information regarding the implementation of these tools, readers are directed to the corresponding references provided in the paper. These references offer specific insights into the deployment process, including the methodologies and techniques used by each institution.

It is noteworthy that the tools used for deployment in these examples are publicly available on GitHub, a platform for sharing and collaborating on open-source projects. By adopting open-source practices, the institutions promote transparency and encourage knowledge exchange within the scientific and medical communities. The availability of these tools to the public enables researchers and clinicians from various institutions to leverage and build upon the existing work, facilitating further advancements in the field of AI in health care.

Through these examples, we want to show the readers that even if some AI tools are not commercially available, creating an AI deployment using open-source tools like MONAI Deploy and following the imaging standards is possible.

### Pediatric Bone-Age Determination

Bone age is a valuable metric of skeletal maturity in pediatric and adolescent patients. It is normally performed by an experienced radiologist who manually compares the bones on a frontal x-ray of the hand and wrist against a decades-old atlas. This is a time-consuming process that must be performed by a radiologist with related expertise yet remains prone to interpreter variability.

**Figure 2:**
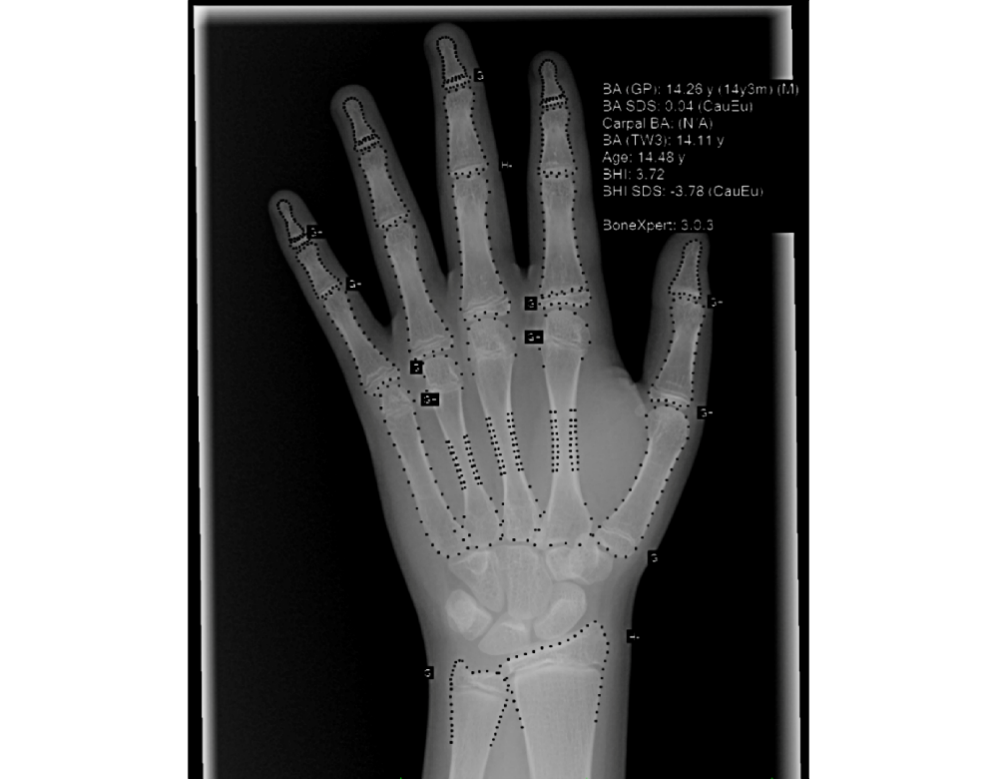
Output from the artificial intelligence bone-age application showing identified features and quantitative results. BA: bone age, averaged over the 21 tubular bones; BHI: Bone Health Index; CauEuN: European Caucasian North population; GP: Greulich-Pyle; SDS: standard deviation score; TW3: Tanner-Whitehouse Version 3.

To address this variability, Guy’s and St Thomas Hospital investigators integrated a commercial AI bone-age application into its clinical workflow (Figure 2). The bone-age application [37] was deployed according to the vendor specification with dedicated hardware running the service, and users were required to manually send imaging studies to the DICOM node when calculating bone age.

Approximately 3 months after deployment, a study was performed to quantify radiologists’ time saved by the application. In fact, no significant time savings were found when using the AI bone-age application versus manual measurement by radiologists. The most likely explanation was that the radiologists had to forward the examination data to the AI system when reporting, thereby nullifying any potential efficiency that the AI system itself introduced. This experience illustrates that the management of clinical workflows and the enablement of imaging AI applications are closely coupled and cannot be considered in isolation.

### Brain Tumor Segmentation and Progression Detection From Longitudinal Magnetic Resonance Imaging Data

The current standard for assessing treatment response for gliomas is based on changes in 2D cross-sectional tumor measurements of anatomical brain magnetic resonance imaging (MRI) [43-45]. However, studies show that 3D volumetric assessments outperform 2D measurements for reliable tumor progression detection [46].

University of California San Francisco investigators deployed a machine learning–based, human-in-the-loop workflow to automatically segment longitudinal low-grade glioma tumors within brain MRI data, compute volumetrics, and generate reports showing tumor volume changes along several time points. This clinical workflow was intended to assist physicians during tumor board meetings in assessing treatment options for patients with low-grade glioma.

**Figure 3:**
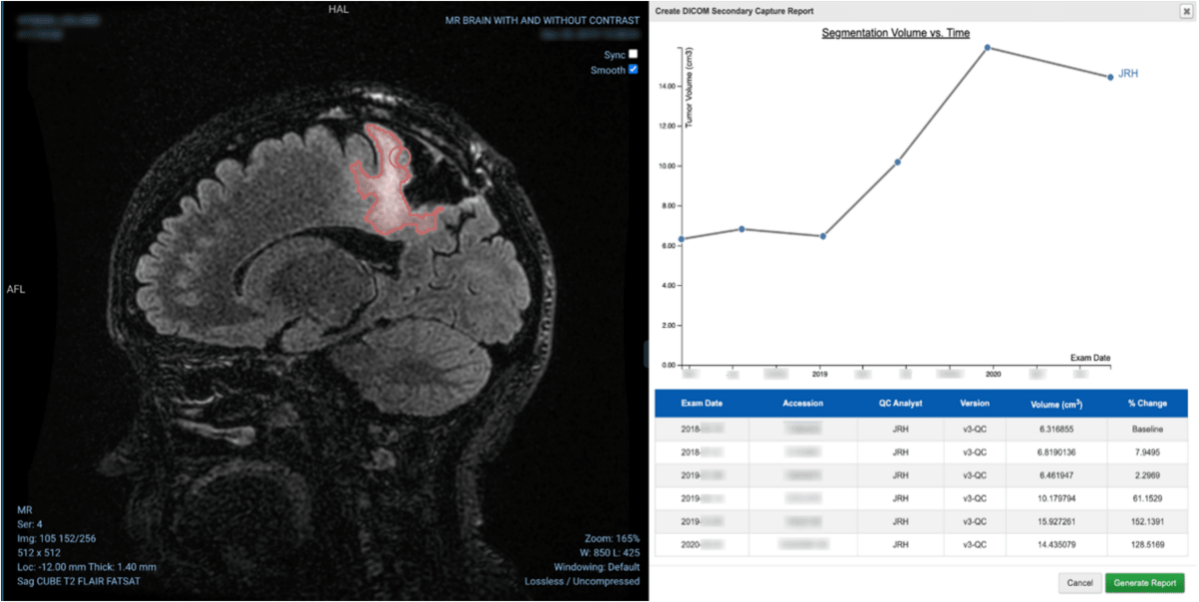
Glioma segmentation (left) and clinical validation of segmentation-based detection of glioma progression (right).

These online interactive and interpretable progression assessments can be presented to radiologists within 10 minutes of DICOM transmission (Figure 3). Automated segmentation has lower variability in volumetric measurement compared with manual segmentation, and performance matches the gold standard under the same acquisition sequence (GE 3D CUBE) [47].

### Radiology Cooperative Network

The Radiology Cooperative Network **(**RACOON) unites all university hospitals in Germany in a nationwide infrastructure [48] that enables federated training and application of AI for medical imaging. As part of the Network of University Medicine [47], the project represents a consortium consisting of the German Cancer Research Center, all 38 German University of Radiology departments, public research institutes, Fraunhofer MEVIS, Technical University Darmstadt, and commercial partners (Mint Medical and ImFusion). RACOON’s hybrid network architecture enables federated analysis through common nodes at all partner sites (RACOON-NODE) complemented by a secure cloud environment that allows the pooling of datasets and hosts central services (RACOON-CENTRAL). Each node provides a toolset for structured reporting [49], image annotation, and segmentation, as well as an open-source platform for AI training and automated image analysis (Joint Imaging Platform) [50].

### Detecting MRI-Hazardous Implanted Leadless Electronic Devices on Radiographs

Particular implanted leadless electronic devices are considered either stringently MRI conditional or MRI unsafe, necessitating restriction to basic MRI or patient exclusion from an MRI examination, respectively. Ideally, the implanted device type is documented in a patient’s electronic health record and available to MRI examination supervisors. Otherwise, radiographs are often used to prescreen patients for such devices and, accordingly, for their eligibility to undergo standard MRI. Unfortunately, such devices can be easily overlooked or misrecognized on x-rays due to their small sizes, similar appearances, or suboptimal image acquisition (eg, insufficient contrast between the device and surrounding tissues). This can lead to serious consequences or injury to the patient during MRI, especially at newer high-field strengths (eg, 7 Tesla).

**Figure 4:**
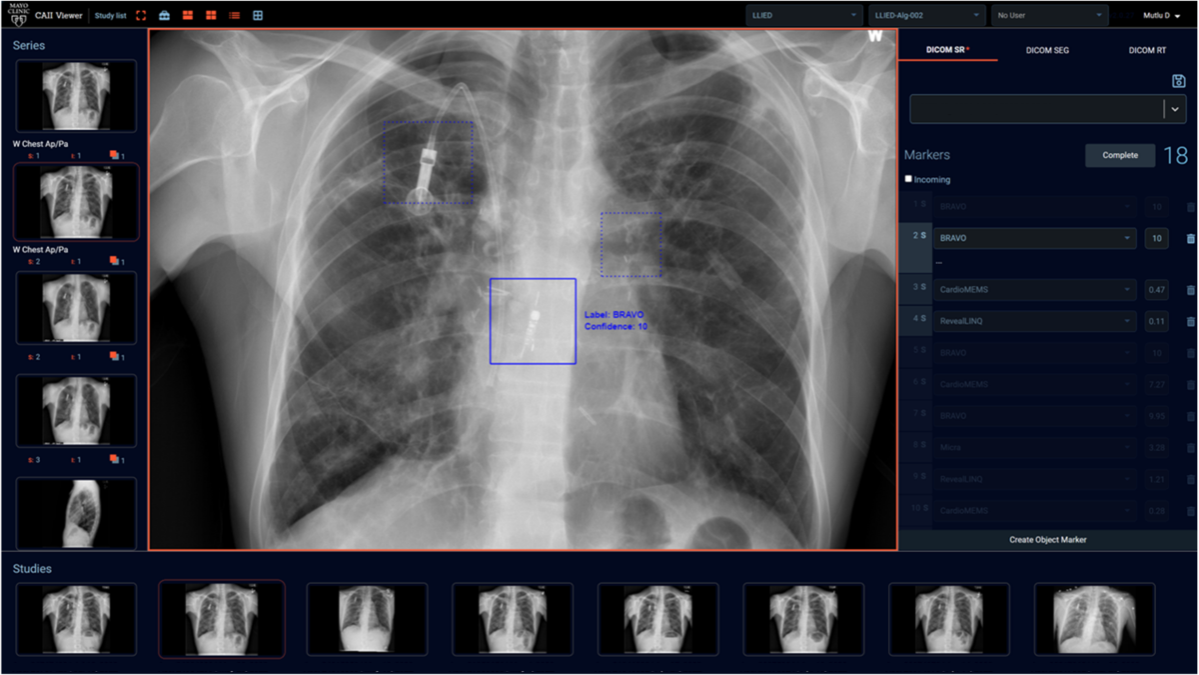
Magnetic resonance imaging–unsafe device (bravo esophageal reflux pH capsule) correctly detected and identified (with 10/10 certainty) on chest X-ray by model inference (shown as a solid bounding box), displayed and adjudicated by a radiologist on the viewer. Two cascading AI models were used, with the results of the first model (for detection based on a faster region-based convolutional neural network) fed into the second model (for identification based on a multiclass convolutional neural network). AI: artificial intelligence.

To address these issues, Mayo Clinic investigators developed, upgraded by continuous learning, and deployed a frontal chest x-ray–based device detection algorithm [51]. MONAI Deploy was used for creating an application package that can perform inference on the incoming DICOM images and produce results compatible with the DICOM structured reporting format. Such capability enables the results to be viewed by a clinical viewer (Figure 4).

## Discussion

AI in health care and medical imaging is still in its infancy, despite extensive publishing on state-of-the-art AI models. In addition, to our knowledge, there are few AI applications that can yet prove time savings to radiologist workflows. Measuring efficiency would only be possible by implementing standards, such as the IHE standard log of events [52], in addition to careful user-interface designs to enable users to provide feedback and use the results with minimal workflow impact. Hence, the system and the algorithm performances can be captured, logged, and measured objectively. Such efforts by MONAI Deploy are underway, and demonstrations were made at the IHE Connectathon, 2022, and Radiology Society of North America Imaging AI in Practice, 2022

This report represents an effort specifically by the MONAI-Deploy group of the MONAI Consortium to elucidate the challenges of imaging AI clinical deployment and to guide deployment architectures and solutions to common deployment issues. We delineated the current state of imaging AI implementation within clinical workflows in radiology, discussing the potential barriers and suggesting responses. We believe it is reasonable to expect imaging AI with adequately trained models to aid radiologists in their routine clinical practices; AI is likely to reduce radiologist workloads. However, to incorporate imaging AI into radiology workflows, an infrastructure that efficiently and effectively interfaces with existing operations and infrastructures is essential. If the promise of AI is in making health care, including radiology, more affordable and impactful, such seamless integration is required to deliver on this potential.

This report discussed various integration and workflow requirements for integrating open-source tools for AI algorithm development and deployment for medical imaging. While some of these examples fully use MONAI Deploy build capabilities, others have contributed their requirements and the build capabilities along with software packages to the MONAI software libraries and the Model Zoo [53]. Some of these capabilities were demonstrated through real-world examples discussed in this paper. Most of the capabilities discussed in the real-world examples are contributed to the open-source community through MONAI Deploy. We have showcased these capabilities through real-world use cases. We believe such examples will instill empowerment in hospitals so that they can grow and deploy their in-house tools with community support.

## Conclusions

This paper has examined the current state of community-driven AI deployment in radiological imaging, exploring challenges, integration points, and AI applications through case studies from leading institutions. Our analysis highlights the critical importance of seamless integration with existing clinical workflows, interoperability with health care systems, and adherence to industry standards for successful AI adoption in radiology. Open-source initiatives like MONAI Deploy are empowering institutions to develop and implement their own AI tools, fostering innovation and knowledge exchange. However, rigorous validation of AI models in clinical settings remains a significant challenge requiring collaborative efforts and standardized frameworks. While AI in radiology has made considerable progress, it is still in its early stages. Future work should focus on quantifying efficiency improvements, enhancing user interfaces, and developing robust feedback mechanisms. Ongoing collaboration among researchers, clinicians, and informatics specialists will be crucial to realizing AI’s full potential in enhancing radiological practice and patient care. As these efforts continue, we anticipate more widespread adoption of AI tools in radiology workflows, leading to more efficient, accurate, and patient-centered care. The future of radiology lies in the successful integration of human expertise and AI, promising to revolutionize medical imaging and improve patient outcomes.

## Abbreviations

AI: artificial intelligence
CT: computed tomography
DICOM: Digital Imaging and Communications in Medicine
FDA: Food and Drug Administration
HL7: Health Level 7
IHE: Integrating into the Healthcare Enterprise
MONAI: Medical Open Network for Artificial Intelligence
MRI: magnetic resonance imaging
PACS: Picture Archiving and Communication Systems
RACOON: Radiology Cooperative Network
